# Public service motivation as a mediator of the relationship between job stress and presenteeism: a cross-sectional study from Chinese public hospitals

**DOI:** 10.1186/s12913-019-4483-5

**Published:** 2019-09-03

**Authors:** Jianwei Deng, Yaxin Li, Yangyang Sun, Run Lei, Tianan Yang

**Affiliations:** 1 School of Management and Economics, Beijing Institute of Technology, No. 5 South Zhongguancun Street, Beijing, 100081 People’s Republic of China; 2 Sustainable Development Research Institute for Economy and Society of Beijing, No. 5 South Zhongguancun Street, Beijing, 100081 China; 3 Chair of Sport and Health Management, School of Management, Technical University of Munich, Uptown Munich Campus D, Georg-Brauchle-Ring 60/62, 80992 Munich, Germany

**Keywords:** Challenge stress, Hindrance stress, PSM, Presenteeism, Healthcare service, Big data

## Abstract

**Background:**

Job stress is a strong indicator of presenteeism, but few studies have examined its diverse effects and mediators on presenteeism. This study explored the relationships between job stress, public service motivation (PSM) and presenteeism and how job stress and PSM influence presenteeism in a large national sample of Chinese healthcare workers.

**Methods:**

A cross-sectional survey including 1392 healthcare workers from 11 Class A tertiary hospitals in eastern, central and western China was used in the analysis. Descriptive statistical analysis, correlation analysis and structural equation modeling were used to test the research hypothesis.

**Results:**

Hindrance stress was inversely associated with PSM (β = − 0.27; *P* < 0.001) but significantly positively associated with presenteeism (β = 0.35; *P* < 0.001). PSM was directly inversely associated with presenteeism (β = − 0.35; P < 0.001). PSM partially mediated the relation of hindrance stress with presenteeism.

**Conclusions:**

The findings suggest that efforts to prevent presenteeism among healthcare workers in China should emphasize PSM improvement and reduction of hindrance stress.

## Background

Presenteeism refers to the “potential productivity loss in the workplace due to health and other events” [1]. Presenteeism has been a focus of organizational and human resource management research, because scholars are beginning to realize that presenteeism is a hidden but significant drain on productivity [2] and that it has a greater effect than actual absence on the overall productivity of an organization. Although presenteeism is clearly a substantial financial problem for organizations [3], few studies have examined its antecedents and mechanisms. Job stress is thought to be the main indicator of presenteeism, as it leads to presenteeism by adversely affecting the physical condition of employees [4–6].

Job stress is usually assumed to include multiple dimensions. Cavanaugh et al. reported that challenge stress and hindrance stress underlay scores on items from several popular measures of stress. Challenge stress was viewed by managers as obstacles to be overcome in order to learn and achieve, including high workload, time pressure, job scope and high responsibility. Hindrance stress included stressful demands viewed by managers as unnecessary impediments to personal growth and goal attainment, such as organizational politics, red tape, role ambiguity and concerns about job security [7]. Different types of job stress are usually considered to have diverse effects on productivity-related outcomes [1]. However, existing studies usually analyzed job stress as an aggregate variable and few have assessed how different types of stress affect presenteeism. Thus, we explored how challenge stress and hindrance stress differentially affect presenteeism.

In addition to affecting the physical condition of employees, job stress may lead to presenteeism by affecting employees’ psychological condition, including their public service motivation (PSM). Broadly speaking, PSM is “an individual’s predisposition to respond to motives grounded primarily or uniquely in public institutions and organizations” [8]. A commitment to the public interest, service to others, engagement in prosocial behavior and self-sacrifice underlie the understanding of PSM [8–10]. On the one hand, some common stressors (e.g., red tape, leadership style and other organizational factors) are considered to be important antecedents of PSM [11–13]. Although people become insensitive to others (e.g., by decreased helping and recognition of individual differences and increased aggression) as job stress increases [14, 15], the effects of various types of job stress differ. In a meta-analysis, Lepine et al. tested the impact of different types of job stress and found that challenge stress was associated with high motivation, because people were likely to believe that there is a positive relationship between effort expended on coping with these demands and the likelihood of meeting the demands. Moreover, they were likely to believe that if these demands were met, valued outcomes would occur. However, hindrance stressors were associated with low motivation, because people were unlikely to believe in the presence of a relationship between effort expended on coping with these demands and the likelihood of meeting them [16]. Later, Deng et al. reported that challenge stress was significantly positively associated with PSM, while hindrance stress had adverse effects [17]. On the other hand, PSM might determine the way in which we conceptualize things, which shapes our desires and actions [18]. This would lead to a huge potential loss in productivity, or presenteeism, if PSM was not seriously considered in public-sector employees. Simone et al. examined Spanish civil servants and found that PSM was significantly positively associated with work engagement, which could partly prevent presenteeism [19]. Thus, PSM is important in the relationship between job stress and presenteeism [1, 20].

Person-organization fit theory might further explain the mediating effect of PSM on the relation between job stress and presenteeism. The study of PSM is closed related to the person-organization fit theory, which holds that individual psychology and behavior result from the interaction between persons and organizations. PSM assumes that civil servants are committed to serving the public [10]. Healthcare workers in China prefer to work in the public sector and usually exhibit strong work ability and job performance at the beginning of their employment, when PSM is high and organizations fit them well. However, organizational changes could cause friction between the worker and organization. When employees are in a work environment with high job demands and stress, the imbalance between the individual and organization could alter their PSM and even cause considerable productivity loss, or presenteeism [21, 22].

We targeted Chinese healthcare workers in public hospitals in this study because they provide most health services in China [23] and suffer from long working hours, a high-intensity work pattern and poor physician-patient relations [24–26]. These factors typically cause Chinese healthcare workers to have high job stress and presenteeism [27]. Additionally, public hospitals are an important part of the public sector in China. Employees in public sectors are usually considered to have high PSM [28, 29].

**Fig. 1:**
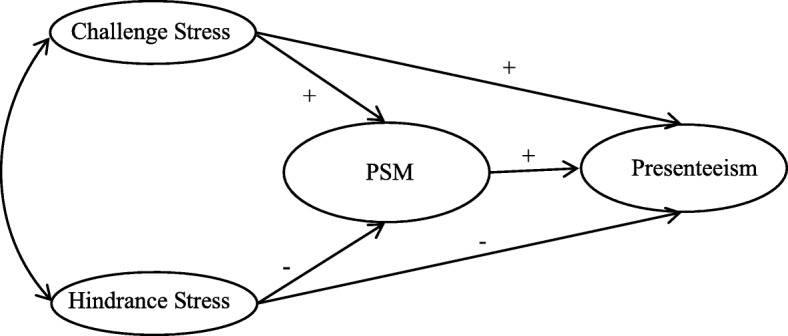
Proposed model of how challenge stress, hindrance stress, and PSM affect presenteeism

Overall, this study focuses on the relationship between job stress, PSM and presenteeism. It examines the mediating effect of PSM on the association between job stress and presenteeism and the effects of different types of job stress on PSM and presenteeism (Fig. 1). This study has potential theoretical and practical contributions. First, it investigated the mediating effects of PSM on the relationship between job stress and presenteeism, which contributes to research on mediators between job stress and presenteeism. Second, the present study yielded practical information on the differential effects of various types of job stress on PSM and presenteeism [17, 27]. Third, it provides empirical evidence regarding the effects of PSM on other psychological constructs and work-related outcomes [30, 31]. Finally, this study provides useful information on how managers of public hospitals seeking to reduce medical costs and improve healthcare quality might effectively motivate and improve the performance of healthcare workers.

## Methods

### Sample

This cross-sectional study was conducted in 11 random-selected representative Class A tertiary hospitals in eastern (*n* = 5), central (*n* = 3) and western (n = 3) China in 2016, in accordance with the ratios of the number of Class A tertiary hospitals in these three regions (5.2: 3.6: 2.6). Ethics approval was received from an independent research ethics committee in China. Because most healthcare services in China are provided by public hospitals and because Class A tertiary hospitals (more than 1000 employees and more than 500 beds) provide the highest level of healthcare services in the country, we included these hospitals in this study. Each study participant provided informed consent. Questionnaires were dispatched on the spot and collected after a pre-specified time to ensure comparability. After random sampling by geographical area and employee ID, we identified and ultimately analyzed data from 1392 participants (including doctors, nurses, pharmacists, medical technicians, and administrative personnel in hospitals and clinics; response rate 97.5%).

### Assessment tools

#### Job stress scale

Job stress was measured with the Challenge and Hindrance-related Self-reported Stress (C-HSS) scale of 11 items, which has been widely used and has been proven to have good reliability and validity [7, 17]. Six items were used to measure challenge stress, and five items were used for hindrance stress. For example, the item, “The number of jobs (or tasks, projects) I undertake”, asks respondents to rate their challenge stress and hindrance stress on a 5-point Likert scale (1 = no stress; 5 = great stress). Higher values reflect greater job stress. In our research, the C-HSS scale was shown to have high reliability (α = 0.91–0.83).

#### PSM scale

PSM was measured with a reliable and validated scale developed by Vandenabeel [32] and validated in previous studies [17]. It includes 18 items in five dimensions: attraction for public policy making (two items), public interest (four items), compassion (five items), self-sacrifice (four items) and democratic governance (three items). The item, “I volunteer to contribute to my community selflessly”, asks respondents to rate their importance to the public interest on a scale from 1 (strongly disagree) to 5 (strongly agree). To make the score more consistent and intuitive, we changed the score directionality of three reverse items by subtracting the original scores from 5. Therefore, higher values reflect greater PSM. In the present study, the scale has high reliability (α = 0.836).

#### Presenteeism scale

Presenteeism was assessed with the Perceived Ability to Work Scale (PAWS), a robust and reliable instrument comprising four items measuring perceived productivity loss. The PAWS had acceptable psychometric properties in previous studies and in a national survey of the United States [33, 34]. The item, “When thinking about the mental demands of your job, how do you rate your current ability to meet those demands?” asks respondents to rate their perceived physical ability on a scale from 0 to 10 (0 = cannot currently work at all; 10 = work ability is currently at its lifetime best). The scale has high reliability (α = 0.89) and acceptable psychometric properties. To understand the score more intuitively, we changed score directionality by subtracting the original scores from 10. Therefore, higher scores indicate greater presenteeism.

### Data analysis

This study used SPSS 20.0 and AMOS 20.0 for the statistical analyses, which included data imputation, descriptive analysis, correlation analysis and structural equation modeling (SEM). The SEM analysis was used to investigate the relationships between challenge stress, hindrance stress, PSM and presenteeism. SEM can identify effect relationships among variables, which are classified as direct or indirect [35, 36].

Imputation was conducted to address the missing values using Expectation-Maximization. Finally, we used the Sobel test to examine the significance of mediated effects [37, 38]. We also used correlation analysis to determine the significance of the correlations between challenge stress, hindrance stress, PSM and presenteeism.

Before conducting SEM, we conducted confirmatory factor analysis to confirm that our model fitted the data well. In SEM, the four latent variables were challenge stress, hindrance stress, PSM and presenteeism. The criteria used to evaluate the model were a root mean square error of approximation less than 0.08 and normed fit and comparative fit indices greater than 0.90, which indicate good model fit [39, 40].

## Results

### Description of respondents

Table 1 shows the characteristics of the final sample after excluding participants without any response. Demographic information was missing for a few participants (3.3 to 5.9%), and 21.3% of respondents were men. Only 3.7% of participants were older than 50 years; most (38.6%) were 25 to 30 years of age. Nearly one third of participants (29.4%) had a master’s degree or higher graduate degree, and 41.5% had undergraduate degrees. Half of respondents had less than 5 years of work experience, 22.1% had 6 to 10 years of work experience, and 24.0% had more than 10 years of work experience. Most participants were nurses (42.3%) or clinicians (30.5%). In terms of job title, 53.1% had a junior title, 27.6% had a mid-level title, and 8.6% had a senior title. Pediatrics (18.7%), internal medicine (16.5%) and surgery (16.2%) were the most common departmental affiliations; only 0.9% of participants were in the oncology department.

**Table 1:** Demographic characteristics of participating healthcare workers (*N* = 1392)

Characteristics	Sample(*n* = 1392)	%
Gender		
Male	297	21.3%
Female	1037	74.5%
Age (years)		
~ 25	189	13.6%
25~ 30	538	38.6%
31~ 35	302	21.7%
36~40	128	9.2%
41~50	138	9.9%
51~55	40	2.9%
56~60	8	0.6%
60~	3	0.2%
Post		
Clinician	425	30.5%
Nurse	589	42.3%
Management	119	8.5%
Medical technician	158	11.4%
Pharmacist	25	1.8%
Education		
Undergraduate	53	3.8%
Junior college	295	21.2%
Undergraduate	577	41.5%
Master	299	21.5%
Doctor	110	7.9%
Title		
Trainee	67	4.8%
Junior	739	53.1%
Mid-level	384	27.6%
Senior	120	8.6%
Seniority (years)		
~3	341	24.5%
3~5	355	25.5%
6~10	307	22.1%
11~20	193	13.9%
20~	140	10.1%
Department		
Physician	229	16.5%
Surgeon	226	16.2%
Obstetrics and Gynecology	132	9.5%
Pediatrics	260	18.7%
Chinese Medicine	102	7.3%
Oncology	12	0.9%
Other clinical Departments	84	6.0%
Medical technology	181	13.0%
Administration and Logistics	90	6.5%

From Table 2 it can be seen that the mean for the challenge stress (M = 3.47, SD = 0.87) was higher than that for hindrance stress (M = 2.85, SD = 1.03). Mean PSM score was 3.72 (SD = 0.94). The average level for presenteeism was 2.59 (SD = 1.68).

**Table 2:** Pearson Correlations between Presenteeism, Stress, and PSM

	Mean	SD	1	2	3	4
Challenge stress	3.47	0.87	1			
Hindrance stress	2.85	1.03	0.532**	1		
PSM	3.72	0.94	−0.077**	−0.225**	1	
Presenteeism	2.59	1.68	0.196**	0.315**	−0.353**	1

Notes. **p < 0.01

### Correlations among study variables

Table 2 displays the correlations between challenge stress, hindrance stress, PSM and presenteeism. The correlation coefficients (r) for items within the same construct (Table 2) were positively correlated. PSM was significantly negatively correlated with challenge stress (β = − 0.077, *p* < 0.01) and hindrance stress (β = − 0.025, p < 0.01), and presenteeism was significantly positively correlated with challenge stress (β = 0.196, p < 0.01) and hindrance stress (β = 0.315, p < 0.01). However, PSM was significantly inversely correlated with presenteeism (β = − 0.353, p < 0.01). Of note, challenge stress was significantly positively correlated with hindrance stress (β = 0.532, p < 0.01).

### SEM

First, we used the Harman Single Factor score to identify common method bias (CMB). All items (measuring latent variables) were loaded into a common factor. When the total variance for a single factor is less than 50%, the data are not likely to have been affected by CMB [41, 42]. We performed the Harman Single Factor test and found that the newly introduced single factor accounted for 24.38% of the variance, which is below the threshold for common method bias (.50). Therefore, we conclude that there was no common method bias in the data.

**Fig. 2:**
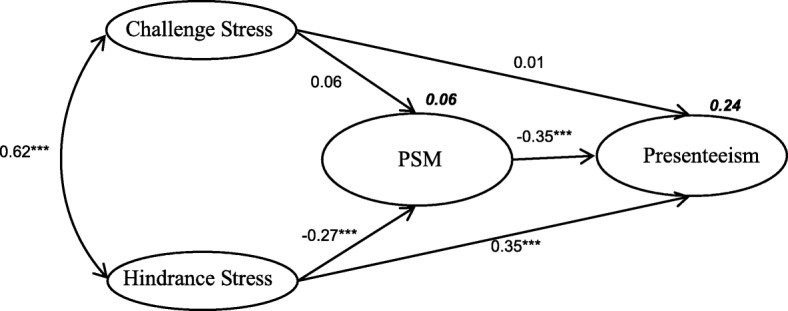
Final model illustrating how challenge stress and hindrance stress influence PSM and presenteeism (*Notes.* *** *p* < 0.001). (numbers not in bold are standardized regression coefficients and numbers in bold explain variability; chi square, 1381.091; degrees of freedom, 164, *p*  < 0.001; root mean square error of approximation, 0.073; normed fit index, 0.917; comparative fit index, 0.926; *** *p*  <  0.001)

Then, analysis of the measurement model showed that our model fitted the data well because the values for the goodness-of-fit and comparative fit indices of each measurement model were all between 0.904 and 0.926. The chi-square values (degrees of freedom and *p*-values) for the measurement model of challenge stress, hindrance stress, PSM and presenteeism were 375.678 (9, *p* < 0.001), 61.361 (5, p < 0.001), 353.728 (5, p < 0.001) and 32.30 (2, p < 0.001), respectively. In the final model (Fig. 2), PSM was directly inversely associated with presenteeism (β = − 0.35; *P* < 0.001). Hindrance stress was negatively associated with PSM (β = − 0.27; P < 0.001) but significantly positively associated with presenteeism (β = 0.35; P < 0.001). However, the path from challenge stress to PSM (β = 0.06; *P* = 0.16) and presenteeism (β = 0.01; *P* = 0.76) was not significant. There was a direct positive association between challenge stress and hindrance stress (β = 0.62; P < 0.001). Challenge stress and hindrance stress explained 6% of the variability in PSM. Challenge stress, hindrance stress and PSM explained 24% of variability in presenteeism. Criteria for fitness, such as root mean square error of approximation, goodness-of-fit index, comparative fit index and normed fit index, indicated that the revised model was more appropriate (Fig. 2).

Finally, the indirect effect was only significant between hindrance stress and presenteeism (Sobel z = 5.28; *p* < 0.001), while the indirect effect between challenge stress and presenteeism (Sobel z = − 1.54; *p* = 0.16) was not. The relationship between hindrance stress and presenteeism was partly significantly mediated by PSM.

## Discussion

Job stress is a strong indicator of presenteeism, but previous studies have mostly considered job stress as an aggregate variable and focused on the negative effects of job stress on presenteeism. We also know that job stress not only directly affects presenteeism, but also leads to presenteeism by adversely affecting individual health [4, 27]. Whether job stress leads to presenteeism by affecting the psychological condition of employees is unclear. Therefore, we used national survey data from 1392 Chinese healthcare workers to explore the positive and negative effects of job stress on presenteeism and the mediating effect of PSM on the relationship between job stress and presenteeism, which yielded the following important findings.

First, hindrance stress had a negative effect on PSM and a positive effect on presenteeism, which partially confirms the findings of some previous studies. Most of those studies concluded that hindrance stress would adversely affect individual psychology and behavior, resulting in exhaustion, burnout, boredom, loss of enthusiasm and composure and erosion of motivation to learn and work [43–45]. According to the person-organization fit theory, the degree and type of job stress significantly affect the fit between a person and organization, thus affecting individual psychology and behavior [10]. Hindrance stress forces employees to focus on work context and interpersonal relationships rather than on work duties and service, and to pursue personal interests rather than public interests. Therefore, hindrance stress could cause friction between a person and organization, thereby decreasing PSM and increasing presenteeism.

Second, PSM was significantly negatively correlated with presenteeism. Although very few studies have investigated the effect of PSM on presenteeism, PSM has been linked to several positive work outcomes, such as job involvement, organizational commitment and organizational citizenship behavior [29, 46, 47]. These positive work outcomes indicate greater work ability and better job performance. The present study defined presenteeism from the perspective of productivity loss; thus, people with high PSM might have lower presenteeism. In addition, we can view this result from the perspective of PSM theory, which holds that public-sector employees with high PSM are more likely to have high job performance and provide more work input [48]. In China, healthcare workers in public hospitals are public servants, a group usually considered to have higher PSM and to be more altruistic. Therefore, they are more focused on their work and on overcoming problems related to health, work, family and interpersonal relationships, which would increase productivity and reduce presenteeism.

Third, as expected, PSM mediated the relationship between hindrance stress and presenteeism. Most previous studies regarded health as a main mediating variable between job stress and presenteeism. For example, job stress caused headache, neck pain and strain, thereby increasing presenteeism [49]. However, job stress may also reduce enthusiasm and motivation for work, which would increase presenteeism. The present study provides empirical evidence that PSM partially mediates the effects of hindrance stress and presenteeism. This result shows that in Chinese public hospitals hindrance stress affects healthcare workers’ presenteeism through PSM and other factors. A future study should target PSM as a predictor of presenteeism.

Overall, this study focuses on the relationship between job stress, PSM and presenteeism. Our findings indicate that hindrance stress has a negative effect on PSM and a positive effect on presenteeism. To improve the quality of health service and decrease counterproductive behaviors (presenteeism) among Chinese healthcare workers, the first priority is to reduce job stress, especially hindrance stress. Managers of public hospitals, for example, should create an environment that supports workers by arranging appropriate workloads or reducing red tape and by promoting job security [50]. In addition, because PSM indirectly mediates the association between job stress and presenteeism, its effect on coping with job stress and presenteeism should be considered as part of policy-making and management. Managers of public hospitals should optimize staff recruitment and selection to attract individuals with high PSM [51].

### Limitations

Our study has several limitations. First, all data were self-reported. Recall, personal perception and perceived stress when completing the questionnaire might have affected the accuracy and objectivity of the assessment and could have introduced bias. Future studies should consider both subjective and objective data on job stress, PSM and presenteeism. Second, our conclusions are based on a cross-sectional database, which limits inferences regarding causality. A third limitation is that the research focused on healthcare workers in Class A tertiary hospitals and excluded those in primary and secondary hospitals, which limits the generalizability and robustness of our conclusions. Therefore, in the future, healthcare workers from primary and secondary hospitals should be included in the research, to verify our hypothesis and models. Finally, the psychometric properties of the Chinese versions of the scales used in this study have not been assessed in a large sample. Nevertheless, we feel that these limitations do not invalidate the present conclusions.

## Conclusion

PSM has a mediating role in the association between job stress and presenteeism among hospital employees. Both dimensions of reported job stress (challenge stress and hindrance stress) have adverse effects on PSM. Hindrance stress is positively associated with presenteeism, but challenge stress does not significantly directly affect presenteeism. To improve job performance and service quality among healthcare workers, job stress should be reduced and PSM increased. Managers of public hospitals should create an environment for workers that limits worker stress and reduces presenteeism.

## Data Availability

The datasets used and/or analyzed during the current study are available from the corresponding author on reasonable request.

## Abbreviations

C-HSS: Challenge and Hindrance-related Self-reported Stress
PAWS: Perceived Ability to Work Scale
PSM: Public Service Motivation
SEM: Structural Equation Modelling
